# Conceptualising engagement with HIV care for people on treatment: the Indicators of HIV Care and AntiRetroviral Engagement (InCARE) Framework

**DOI:** 10.1186/s12913-023-09433-4

**Published:** 2023-05-04

**Authors:** Claire M. Keene, Jonathan Euvrard, K . Rivet Amico, Ayesha Ragunathan, Mike English, Jacob McKnight, Catherine Orrell, the InCARE Stakeholder Group, Anna Grimsrud, Beth Harley, Erin von der Heyden, Ingrid Eshun-Wilson, Ingrid Katz, Kirsten Arendse, Laura Beres, Michael Mugavero, Tali Cassidy, Tamsin Phillips

**Affiliations:** 1 Oxford Centre for Global Health Research, Nuffield Department of Medicine, University of Oxford, Oxford, UK; 2 Centre for Infectious Disease Epidemiology and Research, School of Public Health and Family Medicine, Faculty of Health Sciences, University of Cape Town, Cape Town, South Africa; 3 Health Behaviour and Health Education, School of Public Health, University of Michigan, Ann Arbor, MI USA; 4 Department of Medicine, Faculty of Health Sciences, University of Cape Town, Cape Town, South Africa

**Keywords:** Adherence, Antiretroviral therapy, Engagement, HIV care continuum, Retention, Self-management

## Abstract

**Background:**

As the crisis-based approach to HIV care evolves to chronic disease management, supporting ongoing engagement with HIV care is increasingly important to achieve long-term treatment success. However, ‘engagement’ is a complex concept and ambiguous definitions limit its evaluation. To guide engagement evaluation and development of interventions to improve HIV outcomes, we sought to identify critical, measurable dimensions of engagement with HIV care for people on treatment from a health service-delivery perspective.

**Methods:**

We used a pragmatic, iterative approach to develop a framework, combining insights from researcher experience, a narrative literature review, framework mapping, expert stakeholder input and a formal scoping review of engagement measures. These inputs helped to refine the inclusion and definition of important elements of engagement behaviour that could be evaluated by the health system.

**Results:**

The final framework presents engagement with HIV care as a dynamic behaviour that people practice rather than an individual characteristic or permanent state, so that people can be variably engaged at different points in their treatment journey. Engagement with HIV care for those on treatment is represented by three measurable dimensions: ‘retention’ (interaction with health services), ‘adherence’ (pill-taking behaviour), and ‘active self-management’ (ownership and self-management of care). Engagement is the product of wider contextual, health system and personal factors, and engagement in all dimensions facilitates successful treatment outcomes, such as virologic suppression and good health. While retention and adherence together may lead to treatment success at a particular point, this framework hypothesises that active self-management sustains treatment success over time. Thus, evaluation of all three core dimensions is crucial to realise the individual, societal and public health benefits of antiretroviral treatment programmes.

**Conclusions:**

This framework distils a complex concept into three core, measurable dimensions critical for the maintenance of engagement. It characterises elements that the system might assess to evaluate engagement more comprehensively at individual and programmatic levels, and suggests that active self-management is an important consideration to support lifelong optimal engagement. This framework could be helpful in practice to guide the development of more nuanced interventions that improve long-term treatment success and help maintain momentum in controlling a changing epidemic.

**Supplementary Information:**

The online version contains supplementary material available at 10.1186/s12913-023-09433-4.

## Background

### Engagement is increasingly important as the public health response to the HIV epidemic evolves

The HIV epidemic has been one of the most influential in the last century, with devastating economic, social and health consequences [1]. However, with increasing success in providing access to antiretroviral treatment (ART), HIV has evolved from an acute illness with inevitable mortality to a chronic disease that is managed by millions of patients as a normal part of their life and community [2]. As the ‘lower hanging fruit’ of access is achieved [3], the goals of ‘success’ have shifted from a focus on diagnosis and linkage to services, to long-term maintenance of treatment success for those on ART [4].

This shift in focus to long-term treatment success has changed the health system’s appreciation of the patient’s role in ART management, valuing patient ownership of their medication management and of their interactions with the health system [5]. It also puts pressure on health systems to make long-term investments in treatment and service delivery that support sustained adherence to ART [6]. There is increasing recognition of the need for health system delivery of people-centred services to support consistent, high levels of engagement as a route to maintaining treatment success [7-9]. This puts the health system’s support of optimal engagement at the “top of the list of policy priorities” [10] to facilitate the benefits of accessible treatment in controlling the epidemic [11-14] and maintain the public health gains that scaling ART has delivered [15].

### Engagement is a complex concept

Long-term engagement with care is a fluctuating and dynamic process [16]. Initiating ART represents a ‘biographical disruption’[17] where daily life and its meaning, relationships, social networks and plans for the future are upset and need to be recalibrated [18]. Once on established treatment, people continue to move in and out of the system over their treatment journey [19, 20] as factors in their life shift and change [21]. The treatment journey requires navigation of a medically and socially complex condition, negotiation of often unrealistic healthcare worker expectations, coping with social and internal stigma and management of the health system’s demands for the exceptional levels of engagement needed to achieve treatment success – all while balancing their treatment with the competing priorities in their often challenging day-to-day lives [22].

Engagement with HIV care is also complicated by societal demands that are not present in non-communicable chronic diseases: sustaining virologic suppression prevents onwards transmission, making engagement with care a ‘pro-social behaviour’ and a moral obligation [22]. This places an added burden of treatment success directly on the patient in addition to their own health goals, despite myriad personal, health system and contextual factors influencing patient engagement and tipping them towards or away from engagement with care [21].

The shift in conceptualising HIV as a chronic condition over the past 20 years has been helpful to re-orient HIV services and research. However, the conventional understanding of ‘chronic disease’ as stable, manageable, asymptomatic and linked to lifestyle, fails to capture the experience of people living with HIV [6]. The available chronic disease language’s inadequacy in describing the realities of people’s relationship with HIV adds to the complexity of articulating an understanding of life-long HIV care engagement [6].

### Engagement is not a well-defined concept

The term ‘engagement’ is often not explicitly defined in healthcare literature [23], and is used loosely in HIV-specific literature to refer to a broad spectrum of ‘engagements’ with the health system: linkage to care [24], interaction with or use of services [25-27], appointment attendance [28], medication and schedule adherence [29], active participation in care [30, 31] or patient management of their condition [32]. It is often used in the context of the Joint United Nations Programme on HIV/AIDS (UNAIDS) 95–95-95 targets (aim to have 95% of people living with HIV know their status, 95% of those on ART and 95% of those virologically suppressed by 2030 [33]) and the cascade of HIV care (diagnosis, linkage, retention and virologic suppression) [34].

While these concepts are interrelated [7] and often used interchangeably [35], each has different implications for intervention and strategy decisions [16]. Evaluations often focus on one specific dimension, such as retention or adherence, and so do not account for the complex, multi-dimensional, dynamic process of engagement [23]. Lack of clear definitions also hamper comparable measurement of engagement with HIV care [36], making it more difficult to compare programmes or interventions and make evidenced-based decisions [37]. This is particularly pertinent for less commonly evaluated dimensions such as active participation or self-management, which are not routinely measured. This results in little evidence on their use and a lack of data to clarify their definition and their relationship to ‘engagement’. This loop reinforcing the lack of data will require an iterative process of incremental gains in clarity, which is essential if health services are to operationalise the concept of engagement to facilitate sustained HIV treatment success [23].

To address the demands for more nuanced definitions of engagement [23] and the ambiguity around this crucial concept that limits its evaluation, we developed a pragmatic framework to describe engagement with HIV care for people on ART.

## Methods

The definition of the term ‘engagement’ is often assumed to be commonly understood in literature. The clarification of such ‘taken for granted’ concepts is gaining popularity to challenge how we think about concepts to inform clinical practice and guide research [23]. We examined the concept of engagement with HIV care from the perspective of the health service’s evaluation of engagement, to guide clinical management and implementation of interventions to improve outcomes. While Morse’s Pragmatic Utility approach was not explicitly used, in line with the approach’s focus on the usefulness of a concept in practice [38] this framework was initially developed with a specific purpose: to inform the search and analysis stages of a systematic scoping study of measures of engagement with HIV care, by identifying critical, measurable dimensions of engagement from a health service-delivery perspective to guide evaluation [39]. However, it was found by stakeholders to be useful more broadly in making sense of the complex concept of engagement with HIV care and to articulate an understanding that could potentially be useful in practice.

### Framework development

**Fig. 1:**
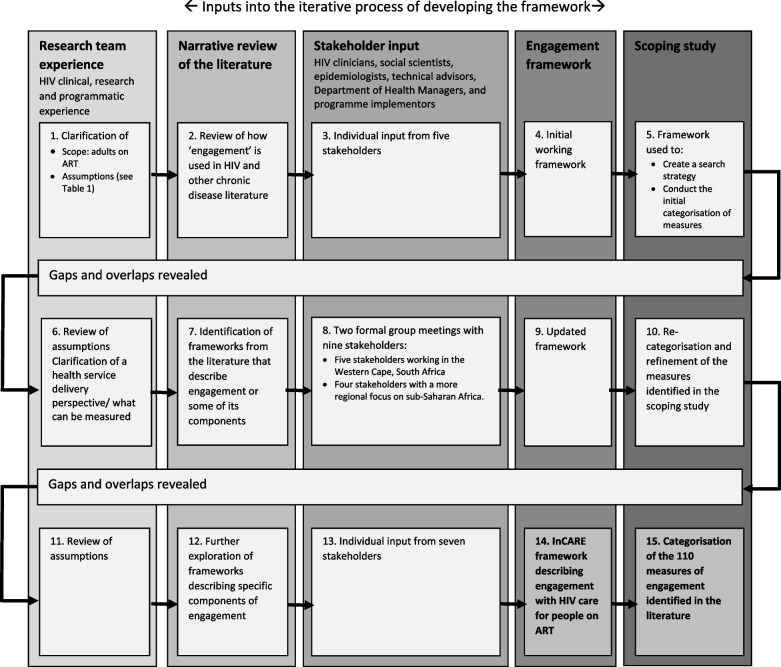
Iterative process of refining the engagement framework and categorisation of measures of engagement

The process of framework development was pragmatic and iterative. Three broad rounds drew on multiple inputs: the research team’s experience, a narrative literature review and framework mapping, a process of expert stakeholder engagement and a process of identifying and categorising measures of engagement scoped from the literature in a formal scoping study (Fig. 1). The initial set of engagement dimensions was iteratively refined to produce the framework presented below.

The research team had experience in clinical management of HIV patients as well as programme and health system development and evaluation for HIV and other conditions. The narrative review identified a number of definitions of engagement with HIV care such as Bright et al.’s definition of engagement [23]. It also identified existing frameworks that contributed to the process, including: the UNAIDS 95–95-95 cascade [33], the concept of the ‘revolving door’ of HIV care retention [34], Theories of Practice applied to HIV engagement [10], the Chronic Care Model [40] and the Situated Information Motivation Behavioral Skills Model of Health Care Initiation and Maintenance model [41]. The narrative review was supplemented by frameworks in related areas such as the Mutuality Framework of Engagement with Pre Exposure Prophylaxis [42] (see Supplementary material Box 1 and Fig. 1 for further information on these definitions and frameworks).

**Fig. 2:**
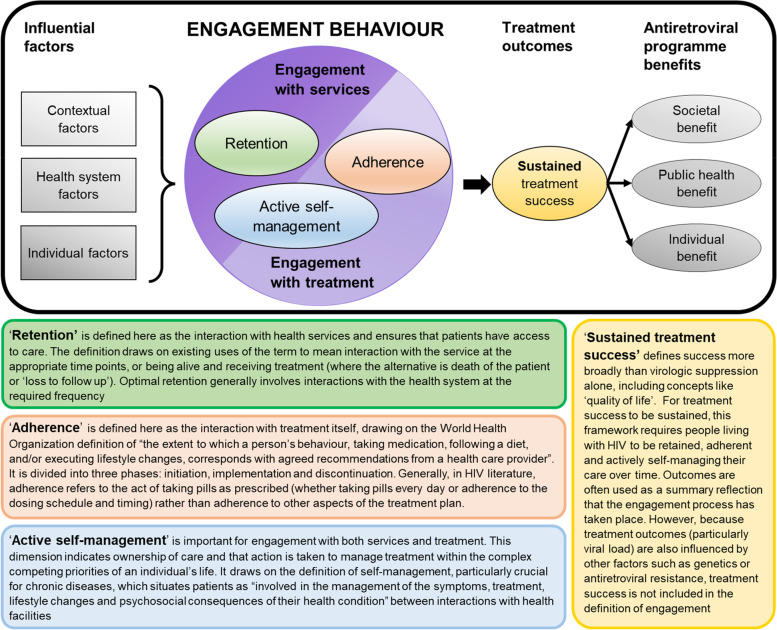
Indicators of HIV Care and Antiretroviral Engagement (InCARE) Framework, drawing from multiple definitions of retention [44-46], adherence [47-49], active self-management [50-52] and treatment outcomes [53-55]

The stakeholder engagement process is described in detail in the Supplementary material (Box 2, Tables 1 and 2, and Figure 2). In short, 13 expert stakeholders (ranging from HIV clinicians to programme managers and academic researchers with experience in sub-Saharan African contexts) were consulted at multiple points in the framework development process to provide input on: the initial assumptions and focus, the framing of engagement with care, distinction between concepts that reflect engagement itself from those that reflect factors influencing it, and the applicability of the framework in practice.

Application of the framework to the search for and categorisation of measures of engagement in a parallel scoping study process, identified gaps in the description of engagement and overlaps in the dimensions. This directed further literature review and stakeholder input. The framework was tested by applying it to a list of 110 measures of engagement identified in the related scoping study to categorise them into mutually exclusive dimensions of engagement with HIV Care [39].

The assumptions and decisions made in developing the initial working definition of engagement are outlined in Table 1.

**Table 1:** Assumptions on engagement and their impact on the development of the initial framework

Assumption	Consequence
Engagement with initiation and maintenance of treatment are conceptually different behaviours [41]. We focused on maintenance of engagement with treatment (those already initiated on ART at any point)	Diagnosis and initiation of ART were not considered in this framework, and rather seen as a separate set of behaviours Re-engagement was considered as part of the dynamic cycle of engagement with treatment once antiretrovirals have been initiated
We focused on adult engagement as adults make up the majority of people living with HIV globally [43]	Dimensions particular to engagement in childhood, such as caregiver relationships, were not explored
We focused on the individual’s contribution to engagement as measured from a health service-delivery perspective	Only dimensions that reflected a person’s engagement behaviour that could be observed by the health system were included The health system’s position in the concept of ‘patient engagement with HIV care’ was not included, and engagement that could not be observed by the health system’s monitoring and evaluation machinery was also not included The patient voice is not incorporated into this framework
Engagement was considered as a set of observable and measurable behaviours, distinct from individual, contextual and health system factors and the success or failure of treatment	Engagement behaviour was placed between the influential factors and the outcome of treatment success, as a separate concept
Engagement was considered with HIV care overall, and not just with healthcare services	Engagement behaviour was divided into ‘engagement with services’ and ‘engagement with treatment’ as separate components, with ‘engagement with treatment’ often taking place outside facility interactions
The working definition of engagement included retention, adherence and an ‘other’ component. The ‘other’ dimension was initially over-inclusively defined, encompassing participation and self-management	The ‘other’ dimension was subsequently refined through the stakeholder engagement and scoping study processes to a dimension labelled ‘active self-management’

## Results

### Proposed definition of engagement

Building on Bright et al.’s definition of engagement [23], the following definition is proposed as a definition of engagement with HIV care for those on ART, from the health service-delivery perspective:

*Engagement is a dynamic state comprising three dimensions: retention in services, adherence to medication and active self-management of care. It incorporates a process of connecting with all aspects of HIV care (services and treatment itself), interacting with the individual as an active, invested collaborator in healthcare and facilitating maintenance of successful treatment outcomes.*

### Indicators of HIV care and antiretroviral engagement: the ‘InCARE’ framework

The developed framework is shown in Fig. 2. It describes engagement for people already on ART as separate from engagement with services to initiate treatment [41]. Engagement with HIV care is a multi-dimensional set of observable behaviours that people practice, rather than a static characteristic of the individual themselves as being an ‘engaged’ or ‘good’ patient. Engagement behaviour is dynamic rather than a permanent state, with people practising variable engagement at different points over the lifelong course of their treatment journey. Engagement with HIV care is reflected by measurable actions that reflect whether a patient is consistently engaging with services and treatment: ‘retention’, ‘adherence’ and ‘active self-management’.

The practice of engagement is separate from the wider contextual, health system and individual factors that are important for engagement, but influence this practice rather than being considered engagement itself [21, 56, 57]. Contextual influences include community dynamics, social resources, socioeconomic status, responsibilities, stigma, social norms, social capital and support, societal demands and politics [21, 22, 58]. Health system organisation covers delivery systems and differentiated model options, supply of medication, health information systems, decision support and relationships with healthcare workers [21, 59]. Individual factors influencing the practice of engagement behaviour include information, motivation and behavioural skills, self-efficacy, acceptance of diagnosis, demographics, clinical state (such as CD4 count) and comorbidities (including mental health and substance abuse amongst others) [10, 21, 41].

Maintenance of engagement with HIV care facilitates and sustains successful treatment outcomes. Treatment success is the ultimate goal of HIV programmes [60, 61], as it facilitates the benefits of ART: individual health benefits of improved health and reduced early mortality [62]; public health benefits including reduced horizontal and vertical transmission [7, 60], reduced costs associated with managing antiretroviral resistant cases and clinically unwell patients [63] and the health system advantages of stable patients currently qualifying for differentiated service delivery models [63]; and societal benefits such as a healthier working age population available to parent their children [1].

## Discussion

Clinical and public health approaches have shifted from treating HIV as an acute emergency to viewing it as a chronic disease: where the patient takes the primary role in the management of their condition and differentiated service delivery models reduce contact with both facilities and providers [5, 53]. This makes maintaining engagement with HIV care more crucial to sustain treatment success [7, 8] and highlights the importance of person-centred support for a person’s lifelong engagement with HIV care [7-9]. We developed a pragmatic framework to conceptualise engagement for people on ART for use by health services to comprehensively evaluate engagement, understand programmes and target interventions to optimise engagement with HIV care.

### Pragmatic utility of the InCARE framework for research, implementing programmes and clinical management

#### Engagement as a behaviour rather than a characteristic of individuals

This framework recognises that maintaining engagement with care is an observable behaviour, distinct from the engagement required to initiate ART [41]. Understanding engagement as a behaviour, rather than a static patient phenotype, shifts the focus away from a dichotomous ‘good’ or ‘bad’ patient categorisation. This normalises the cycle of engagement as people disengage and re-engage with care over time [19, 20]. Suboptimal engagement can then be understood as an expected reality and managed with compassion rather than punished as a failure. This also shifts the health service’s focus to a person-centred approach, facilitating the treatment of patients as active participants in their treatment rather than passive recipients of care who need a paternalistic relationship with the system to enforce engagement [31, 64].

#### Engagement as a dynamic process rather than a linear cascade

The cascade approach to HIV engagement has served as a framework for identifying gaps in coverage and service performance [44]. It has contributed to achieving some of the ‘low hanging fruit’ in the progress of HIV services such as improved standardisation of measures of retention and scale up of ART [10]. However, it has been criticised as overly linear, unidirectional, and for not accounting for the lived realities of the complex patient journey where patients move in and out of care over time [19, 20, 65, 66]. Ehrenkranz et al. proposed a more cyclical cascade to evaluate the retention dimension at a population level more accurately by structuring the cascade to reflect “actual … behaviour” and making the concept of the cycle of disengagement and re-engagement explicit and expected [34]. This approach is well-suited to monitoring national and global programmes [67].

The InCARE framework attempts to develop this further and describes engagement as a dynamic process where patients move in and out of the desired ‘fully engaged’ state [68] depending on their fulfilment of the three dimensions of retention, adherence and active self-management. This conceptualisation focuses on the relationship between elements of the behaviour to complement the cascade approach, which describes the timing and sequence of transitions between states along a cascade. This again normalises the cycle of engagement [19, 20], and helps to direct interventions to periods of time when individuals are require additional support. It also normalises that individuals will be sub-optimally engaged at some point in their treatment journey, and highlights the need for continued monitoring of engagement once people are stable on ART.

#### Engagement as a mediator of influential factors on treatment outcomes

Engagement behaviour is an observable part of a complex process influenced by many layers within and outside of a person’s control: engagement is negotiated between the patient, their experience of HIV and the health system, and their context of competing priorities [10, 41]. The InCARE framework places engagement as distinct from influential individual, contextual and health system factors that influence this behaviour [69]. The framework also distinguishes between engagement behaviour and treatment outcomes such as virologic suppression or health status [54]. This distinction helps to disentangle the effect that changes in influential factors (such as addressing mental health issues, reducing community stigma or extending clinic opening hours) have on the dimensions of engagement, on engagement overall and ultimately on treatment success.

The scale up of ART has resulted in massive public health gains [15], but has shifted the gaps from treatment access to engagement as the key modifier of the success [4] – a much more complicated outcome to achieve. As incremental gains in treatment success become harder to achieve, it becomes more difficult to show efficacy of interventions if only viral outcomes are considered [70]. Recognising engagement and its dimensions as a measurable state on the path to treatment success [4] could help to evaluate the impact of interventions more accurately and develop more nuanced services targeted to leverage a person’s strengths and to meet their needs. It could also help to identify influential factors to intervene on and understand how they ultimately result in improvements in treatment success.

#### Engagement as a comprehensive set of measurable dimensions

The InCARE framework describes engagement as comprised of three measurable dimensions that reflect interactions with both the services and treatment itself: retention, adherence and active self-management. To achieve long-term treatment success, a person must interact with health services to receive ART (retention), take their treatment according to the schedule prescribed by the clinician so that it can work optimally (adherence) and be actively committed to managing their health between infrequent facility visits (active self-management). The InCARE framework situates each dimension as necessary, but not on its own sufficient, to sustain treatment success over time.

While the concepts of retention and adherence are correlated, the InCARE framework places them as distinct dimensions within engagement behaviour, with retention reflecting periodic engagement with healthcare services and adherence reflecting daily engagement with treatment itself. People can be in one state without necessarily being in the other [71]: for example, people are commonly considered ‘retained’ (medication was dispensed and a viral load measured) but not ‘fully adherent’ (viral load is unsuppressed). Conversely, patients may struggle with barriers to attending appointments but manage their daily adherence well [21]. Suboptimal retention or adherence reflect different issues and require different approaches to improve treatment outcomes, thus the differentiation is important.

Treatment success needs to be sustained over a lifetime of ART to prevent drug resistance and facilitate the benefits that ART provides [72, 73]  While retention and adherence may be sufficient to result in treatment success at a particular moment in a person’s treatment journey (reflected for example by a suppressed viral load), both dimensions of engagement are vulnerable to the many shifting individual, contextual and health system factors (and the dynamics between them [69]) which combine to tip people towards or away from optimal engagement with care [21]. Retention and adherence have also been criticised for not fully defining the multi-dimensional “care engagement process” [74, 75].

Maintenance of engagement, and subsequent sustained treatment success, includes an element of ownership, active involvement and self-management of care between facility visits – termed ‘active self-management’ in the InCARE framework. Along with retention and adherence, self-management is seen as essential in lifelong treatment [40], as the health system’s role in achieving ART success is limited to a few interactions with a person over the course of a year and individuals are ultimately responsible for the lifetime task of day-to-day management of their condition (whether they are managing it well or not, they are in fact managing their health) [50]. People are increasingly required to take an active role in their care: to use medication properly, interpret and report symptoms correctly, make decisions on when to seek care, adjust to the new reality of living with HIV (and what this means for them socially and economically), cope with the emotional consequences of their disease, participate in treatment decisions and manage their care to prevent onward transmission [2]. Fully engaged patients are “informed, activated patients in partnership with their physicians”, who can cope with successes, setbacks and living with uncertainty to remain engaged [2].

Retention, adherence and viral suppression measures detect issues late in the disengagement process [74], so examining active self-management may identify patients with a different risk of ‘tipping’ towards poor engagement and so facilitate earlier intervention to maintain treatment success and prevent poor outcomes [13, 76]. Active self-management could also provide some measure of resilience to changing external factors, so that achieving ‘full engagement’ requires more than simply having (retention) and taking (adherence) a pill every day. It requires all three InCARE dimensions, making the case for the important role that active self-management plays in lifelong engagement with HIV care.

Each dimension may have differing relevance for different use cases: programme and population monitoring, research to develop a deeper understanding of how and why patients engage with their treatment to build better services and directing the clinical management of individual patients. Operationalising this understanding of engagement starts with identifying how each dimension is measured for each of these use cases. Conceptualising engagement as three measurable dimensions allows metrics to be categorised by the dimension they evaluate. This could help to disentangle the effect of interventions more granularly than only considering treatment outcomes [70], particularly if the intervention contributes to better engagement in one dimension but is not sufficient to help the patient sustain treatment success.

HIV is a relatively well-researched disease, and despite its devastating effects has had a transformational effect on the ability of health systems in sub-Saharan Africa to manage chronic disease [77]. Many of the health system barriers that reduce service support of engagement for non-communicable disease patients are common to those living with HIV [78]. Retention and adherence strategies in particular are an aspect of HIV care that has been successful in improving outcomes and could be leveraged to improve the care of many other less well-funded chronic diseases, such as hypertension and diabetes, in lower-resource settings [77, 78]. The dimensions described in this manuscript could be translated and adapted to explore engagement with non-communicable chronic disease, supporting research, programme evaluation as well as the development and implementation of interventions to improve engagement with care.

### Strengths and limitations

This framework was developed in an iterative process between defining the dimensions of engagement and categorising measures of engagement with HIV care scoped from the literature [39]. It drew on multiple inputs from literature as well as experienced clinicians and researchers to articulate the synthesis of a more comprehensive understanding of engagement. Expert stakeholders contributed to delineating the dimensions for greater clarity, separating factors that affect engagement (such as self-efficacy and motivation) from engagement behaviour itself, teasing out the active self-management dimension and finding terms to describe this component and the longitudinal nature of the engagement process more clearly. Pragmatism was also a major concern: how framing engagement differently relates to a change in practice was kept at the forefront of discussions.

A systematic process was used to develop this framework, but it may have benefitted from a guiding approach, such as a pragmatic utility concept clarification analysis. It has also not yet resulted in a definitive definition nor an incontrovertible framework, but is rather an incremental step towards greater clarification of the concept. The InCARE framework offers the next step in the exploration of engagement and its importance in individual care and public health. Further work is required to clarify engagement’s role in the facilitation of treatment success and to build evidence on the active self-management dimension.

While retention (measured by interaction with the health system) and adherence (measured by pharmacy refills, antiretroviral concentrations, laboratory results, healthcare worker assessment, pill counts or self-report) are commonly evaluated aspects of engagement behaviour [74], the science of self-management and related concepts is still at an early stage [79] and has not been prioritised in lower-resource settings [72]. Thus, it is not routinely measured and so is not as well understood as retention or adherence. The dimension of active self-management also required the most refinement (with input from expert stakeholders and the results of the scoping study [39]), with concepts like motivation and self-efficacy being removed from this dimension and placed under ‘individual influential factors’ as an ability to engage rather than a reflection of engagement itself. This framework places active-management in a prominent role as one of the three core dimensions of engagement behaviour, but this position is dependent on further work to identify comparable measures of active self-management and address the paucity of evidence of its association with retention, adherence and the maintenance of treatment success.

## Conclusions

Engagement with HIV care is a critical but complex concept. Supporting people to maintain HIV engagement across a lifetime (with so many factors outside the health system’s control) requires more comprehensive health system approaches than those aimed at only providing access to treatment. The programmes that were so successful in scaling up access to treatment now face a new challenge in adapting their response to the evolving needs of people on ART: developing more person-centred interventions rather than continuing to implement the existing siloed, vertical programmes that work for most people most of the time, but do not accommodate the realities people face in managing their treatment within their specific context [10, 37].

The InCARE framework offers an incremental step towards a more comprehensive and standardised understanding of engagement as a dynamic behaviour. It distils the complex concept of engagement into three measurable dimensions that are pragmatically applicable to both the development of more nuanced interventions, and to characterising elements that the health system could measure for more complete evaluation of engagement at individual and population levels. This framework suggests that active self-management is an important consideration in understanding the maintenance of engagement with HIV care, but further work is needed to build evidence for its importance. As the demands on HIV services become more complex, more thorough understanding and evaluation of engagement with care will be required to support sustained successful treatment outcomes over a lifetime of HIV interactions, and this framework could be useful to advocate for such an approach.

## Supplementary Information

**Additional file 1: Box 1.** Bright et al’s proposed definition of engagement.** Figure 1.** Frameworks reviewed.** Table 1.** Stakeholder list.** Box 2.** Stakeholder engagement process.** Table 2.** Individual survey answers.** Figure 2.** Combined Google Jamboards from both group discussions.

## Data Availability

Data is included as supplementary material. Further data requests can be directed to the corresponding author.

## Abbreviations

ART: Antiretroviral treatment
HIV: Human Immunodeficiency Virus
UNAIDS: United Nations Programme on HIV/AIDS
