# Supplementary material for: Conceptualising engagement with HIV care for people on treatment: the Indicators of HIV Care and AntiRetroviral Engagement (InCARE) Framework
Source: BMC Health Serv Res. 2023 May 4;23:435. doi: 10.1186/s12913-023-09433-4 (PMC10161576; doi:10.1186/s12913-023-09433-4)
Supplement: Supplementary file 1 — Additional file 1: Box 1. Bright et al’s proposed definition of engagement. Figure 1. Frameworks reviewed. Table 1. Stakeholder list. Box 2. Stakeholder engagement process. Table 2. Individual survey answers. Figure 2. Combined Google Jamboards from both group discussions. [file 12913_2023_9433_MOESM1_ESM.docx]

Conceptualising ART patient engagement with HIV care

*Claire M Keene, Jonathan Euvrard, K Rivet Amico, Ayesha Ragunathan, Mike English, Jacob McKnight, Catherine Orrell and the InCARE Stakeholder Group*

Supplementary material

Box 1. Bright et al’s proposed definition of engagement…………………………………………………..………….……2

[Figure 1. Frameworks reviewed 3](#_Toc90387587)

[Table 1. Stakeholder list 4](#_Toc94795266)

Box 2. Stakeholder engagement process……………………………………………….………………………………..…………5

[Table 2. Individual survey answers 6](#_Toc94795267)

[Figure 2. Combined Google Jamboards from both group discussions 7](#_Toc90387588)

References ………………………………………………………………………………………………………………………………………. 8

Box 1. Bright et al’s proposed definition of engagement

Engagement is a co‐constructed process and state. It incorporates a process of gradually connecting with each other and/or a therapeutic program which enables the individual to become an active, committed and invested collaborator in healthcare^1^

##### Mutuality framework

The mutuality framework was developed for women’s engagement with pre-exposure prophylaxis ARVs (PrEP), but lends the concept of different levels of involvement and ownership of the desired behaviour (engaging with PrEP): from distrust (where engagement is avoided), to intermittent engagement (to various degrees in the uncertainty and alignment states) to the other end of the spectrum, mutuality (where patients take ownership of their involvement, require little input from the health services and manage their engagement themselves)^3^. It also frames the patient’s approach to engaging with ARVs rather than characterising the patient themselves, moving away from the dichotomy of ‘good’ and ‘bad’ patients and seeing it as a dynamic spectrum where patients move between states of being over their treatment journey based on their relationship with themselves, the health system and their context^3^. While this framework has not been applied to ARVs as treatment, the concepts resonate with the experiences of patients on ART.

##### Situated Information Motivation Behavioral Skills Model of Health Care Initiation and Maintenance (sIMB-CIM)

The sIMB-CIM applies the components of the Information-Motivation-Behavioral Skills model of health behaviour change to the dynamic concept of engagement in care: framing information, motivation and behavioural skills as driving the maintenance of an established patient behaviour (ongoing decision to remain engaged in care including attendance and adherence) over time^5^. Knowledge interacts with the patient’s motivation and their behavioural skills mediate (the magnitude of the mediating role dependant on the complexity of negotiating engagement in care) the effect on patient engagement behaviour^5^. The model frames engagement as a set of sustained, observable behaviours negotiated between the patient, their experience of HIV and the health system, within their context of competing priorities, limited resources and treatment fatigue^5^. The model understands that sustained patient engagement is the integrated result of the person, situation and context, so the behaviour is “inextricably situated within the cultural, organizational, and structural environment in which they develop and continue to occur”^5^. It also separates initiation and maintenance of engagement^5^


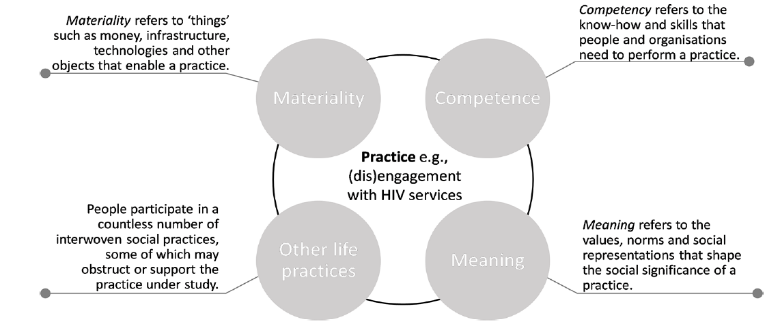

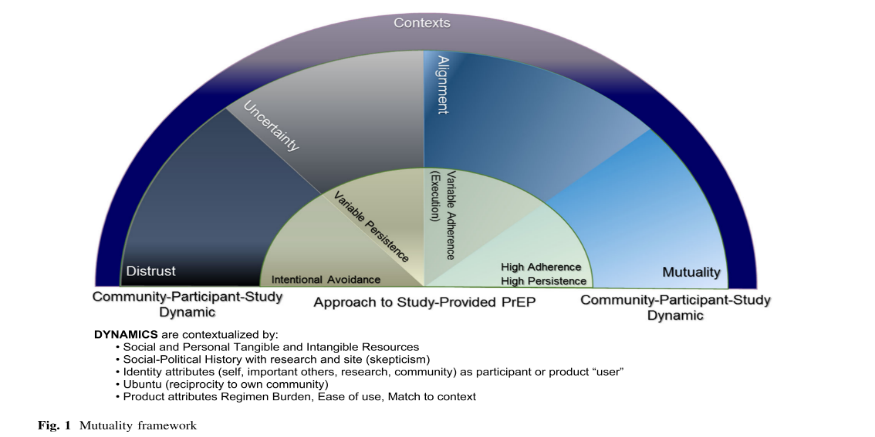

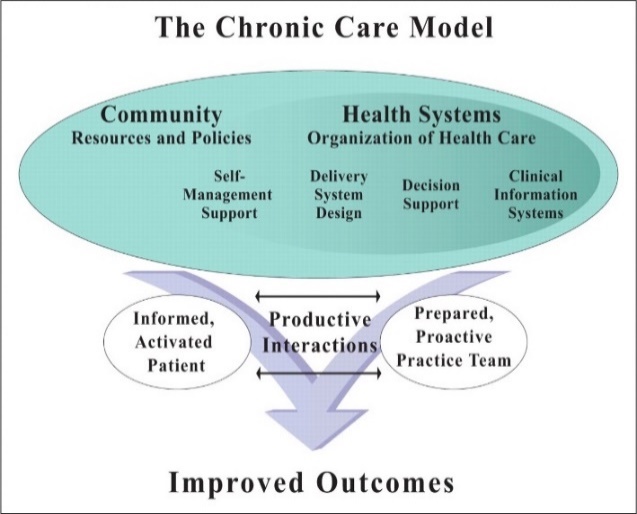


a.

b.

c.

d.


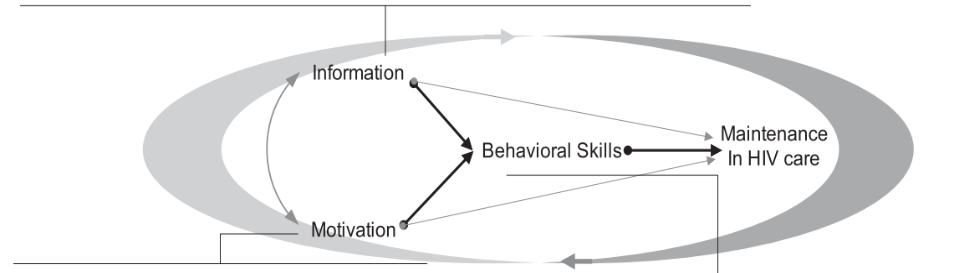


##### Chronic care model and self-management

Recognition of HIV as a chronic disease has significant implications for the care provided, for policy and for the delivery of services, as the goal for chronic healthcare is to “control symptoms and prevent disability rather than cure the disease”^6^, mitigating its impact on the patient^5^. Chronic disease self-management models, seen as critical in non-communicable disease management^6^, have been found to be applicable for PLWH^6^. The patient’s role and responsibility in managing their condition has been increasingly prioritised in chronic disease care. The WHO highlights self-management as important for the health system to support through improving patients’ knowledge, motivation and behavioural skills to encourage patient self-management of their condition^7,8^. This acknowledges that the health system’s role in achieving ART success is limited to a few interactions with patients over the course of a year and patients are ultimately responsible for the lifetime task of day-to-day management of their condition (whether they are managing it well or not, they are in fact managing their health)^9^. It also supports the inclusion of this concept as a key component^8^ of successful chronic disease interventions in the Chronic Care Model^10^, aiming to facilitate patients participating more actively in their care^11^.

##### Theories of practice

Theories of practice have been applied to HIV engagement, separating the practice of engagement behaviours from the factors that influence engagement (materiality, competency and knowledge, meaning ascribed to engagement and other life practices that compete with or support engagement)^2^. HIV engagement is therefore shaped by the dynamic relationships between these elements, which help to understand why patient engagement behaviour differs between individuals and across settings^2^. The practice of engagement is separate from the characteristics of the patient and those factors that are important, but influence this practice rather than being engagement itself^12^

Figure 1. Frameworks reviewed

1. Theories of practice framework applied to engagement with HIV services, taken from Skovdal et al^2^.
2. Mutuality Framework, taken from Amico et al^3^
3. Chronic Care Model, developed by the MacColl Center for Health Care Innovation^4^
4. Situated Information-Motivation Behavioral Skills Model of Health Care Initiation and Maintenance taken from Amico^5^

Table 1. Stakeholder list

|  | Name | Expertise | Dates of engagement |
| --- | --- | --- | --- |
| 1 | **Anna Grimsrud**  PhD, MPH, BSc | Lead Technical Advisor, International AIDS Society | Informal   - 1 February 2021 - 29 July 2021 |
| 2 | **Beth Harley**  MBChB | HIV, AIDS, sexually transmitted infections and tuberculosis (HAST) medical officer for City of Cape Town, South Africa | Local Group   - 13 July 2021 |
| 3 | **Erin von der Heyden**  MBChB, MPH | HIV, AIDS, sexually transmitted infections and tuberculosis (HAST) medical officer for the Khayelitsha and Eastern Sub-structure of the Western Cape Department of Health, South Africa | Informal   - 16 July 2021 - 27 July 2021 |
| 4 | **Ingrid Eshun-Wilson**  MSc, MBChB | Family physician and epidemiologist, School of Medicine, Washington University, United States and University of Stellenbosch, South Africa | Informal   - 16 July 2021 |
| 5 | **Ingrid Katz**  MD, MSc, BA | Physician and Associate Faculty Director, Harvard Global Health Institute, Harvard University, United States of America | Regional Group   - 14 July 2021 |
| 6 | **Jonathan Euvrard**  MPH, MA | Epidemiologist at the Centre for Infectious Disease Epidemiology and Research (CIDER) and PhD student at the University of Cape Town, South Africa | Informal   - Regular discussions   Local Group   - 13 July 2021 |
| 7 | **Kirsten Arendse**  MPhil, MBChB | Programme manager for the ‘Welcome Service’ (differentiated model for patients struggling to engage) for Médecins Sans Frontières’ Khayelitsha project, South Africa | Local Group   - 13 July 2021 |
| 8 | **Laura Beres**  PhD, MPH | Assistant Scientist at Johns Hopkins Bloomberg School of Public Health, Department of International Health, Johns Hopkins, United States of America | Informal   - 20 November 2020   Regional Group   - 14 July 2021 |
| 9 | **Michael Mugavero**  PhD, MSc, MD | Director for the Center for Outcomes Effectiveness Research and Education (COERE), Co-Director of the Center For AIDS Research, and PI of an AHRQ T32 training grant in health services and outcomes research, University of Alabama at Birmingham, United States of America | Informal   - 21 July 2021 |
| 10 | **Rivet Amico**  PhD, BA | Associate Professor, Health Behaviour and Health Education, School of Public Health, University of Michigan, United States of America | Informal   - 1 July 2021 - 15 July 2021 - 21 July 2021   Regional Group   - 14 July 2021 |
| 11 | **Sydney Rosen**  MPA, AB | Research Professor in the Department of Global Health at the Boston University School of Public Health, United States of America and a Co-Director of the Health Economics and Epidemiology Research Office (HE2RO) of the University of the Witwatersrand in Johannesburg, South Africa | Regional Group   - 14 July 2021 |
| 12 | **Tali Cassidy**  PhD, MPH, BSocSci | HIV Epidemiologist for Médecins Sans Frontières’ Khayelitsha project and Division of public health medicine, School of Public Health and Family Medicine, University of Cape Town, South Africa | Informal:   - 22 January 2021   Local Group   - 13 July 2021 |
| 13 | **Tamsin Phillips**  PhD, MPH, B.Optometry | Senior Lecturer, Division of Epidemiology & Biostatistics, School of Public Health & Family Medicine, University of Cape Town, South Africa | Informal:   - 22 January 2021 - 5 August 2021   Local Group   - 13 July 2021 |

Box 2. Stakeholder engagement process

The stakeholders were identified through the study team’s network of contacts and through snowballing. Expert stakeholders were defined as “those people who have knowledge about the topic of concern”^13^. For this scoping study this included those working in the HIV field, ranging from academics to clinical practice, those in practice of development and evaluation of programmes aimed at improving any of the components of engagement and covered a variety of backgrounds (including clinicians, programme managers, social scientists, and epidemiologists). Stakeholders had all conducted research or worked in a sub-Saharan African context, including stakeholders who work in Gugulethu and Khayelitsha where later work for this DPhil will take place.

Stakeholders were consulted at multiple points in the process both in informal discussions and a formalised group process. The aim for the stakeholder interaction for the framework development and scoping study was to collect input from stakeholders to:

1. Review and sense check the framework and the framing of the scoping study results and gather insights on the applicability and platforms for dissemination^14^
2. Contribute to the interpretation of the scoping study results, and build on the evidence by adding a higher level of meaning from their content expertise and perspective^15^
3. Help to identify any key missing sources and measures of engagement for the scoping study^14^

The stakeholder engagement included 13 participants in total, with five providing input before the search, nine providing input on the preliminary analysis (five in the local group and four in the regional group) through group discussions and five providing input on subsequent iterations of the analysis.

#### Informal input

The stakeholder engagement process was used to facilitate input on both the scoping study outputs as well as the framing of the conceptualisation of engagement in a parallel process, and the discussions on both research questions influenced each other in an iterative way. Input was sought from five individual stakeholders (both local and regional) before the search was conducted in order to sense check the framework from which the search terms were developed, then sense check the search terms. Seven stakeholders were consulted after the group meeting to review the adjustments to the framework and categorisation of measures made with the group session recommendations. Input from these one-on-one or small group sessions was captured as summaries of the meeting minutes.

#### Formal group input

The formal group process took place after the preliminary analysis had been completed and drew on nominal group technique; although consensus was not the intention of the discussions and input was rather captured and considered in the final analysis, the nominal group technique’s approach to gathering input was useful in structuring the sessions to answer a particular set of questions^13^:

- Does the framing of engagement make sense?
- Does the way the measures have been categorised make sense?
- Do the identified use cases make sense?

The nominal group process is suited to generating ideas in relation to a particular question, as well as not necessarily needing a clear level of agreement as an endpoint^13^.

The stakeholders on the “expert panel”^16^ was dependent on the availability of the 13 stakeholders identified through the research team’s networks. Two groups of stakeholders were invited to participate: the two smaller groups allowed more space for discussion and input from all stakeholders attending.

Background information, scoping study outputs and focused questions were circulated prior to meeting. In addition, a survey was sent to the local group (as the regionally-focused group were less available for extended input) to ask for individual generation of ideas on ‘what constitutes engagement with care’ and assessment of the categorisation of the groups of measures under the overarching headings. This step drew on the silent generation stage and the voting stage of the nominal group process^13^ as a way for experts to privately form ideas before discussing them with the whole group^16^, and allows each voice to be captured and heard^13^.

Input was captured through an online survey and email responses, which were compiled to inform the focus and areas requiring clarification for the group interaction. The two groups met online and the preliminary findings were presented^15^, with focus on the categories with little agreement from the individual input. The input requested from the group discussions focused on discussions around the engagement framework and sense checking the categorisation of the measures and the identified use cases. The primary author facilitated discussions to capture input from the stakeholders, which was captured on an interactive Google Jamboard. Meeting notes were also taken for both discussions. The disagreements and suggestions were considered in refining the analysis, which is presented in the results.

Stakeholders also contributed to the final manuscript

Table 2. Individual survey answers

| Date of survey | What is engagement? | Definitely is engagement | Unsure if this fits as an element of engagement | Any comments on the elements of engagement and the proposed conceptual framework? |
| --- | --- | --- | --- | --- |
| 2021-07-12 | Any attempt by patient to take charge or act in the interest of their health by interacting with the healthcare system | All dimensions |  |  |
| 2021-07-08 | I understand engagement to be the combination of accessing services when needed/coming to the clinic/picking up your drugs and actually taking your treatment daily as intended. I also think there is an important element of acceptance and autonomy for a patient to be fully engaged. | All dimensions |  | I really love this conceptual framework and think it does a great job of capturing the various elements. One thing I thought of, which may come under motivation or may be completely left field, is considering individual engagement as it relates to others in the individuals life and their social responsibility/expectations. |
| 2021-07-08 | Engagement evokes a positive energy toward something which results in conscious and willing interaction | Participation and self-management | Retention and adherence | Obstacles are important for engagement to continue; given enough obstacles anyone will be pushed to a point of disengagement. So possibly this needs to measured as a separate "entity" to understand engagement in context? |
| 2021-07-05 | Patient engagement with HIV care describes a patient's interaction with their HIV and its therapeutic requirements in partnership with the health services. | All dimensions |  | Missing: spiritual dimension |

Figure 2. Product of the stakeholder engagement process: combined Google Jamboards from both group discussions


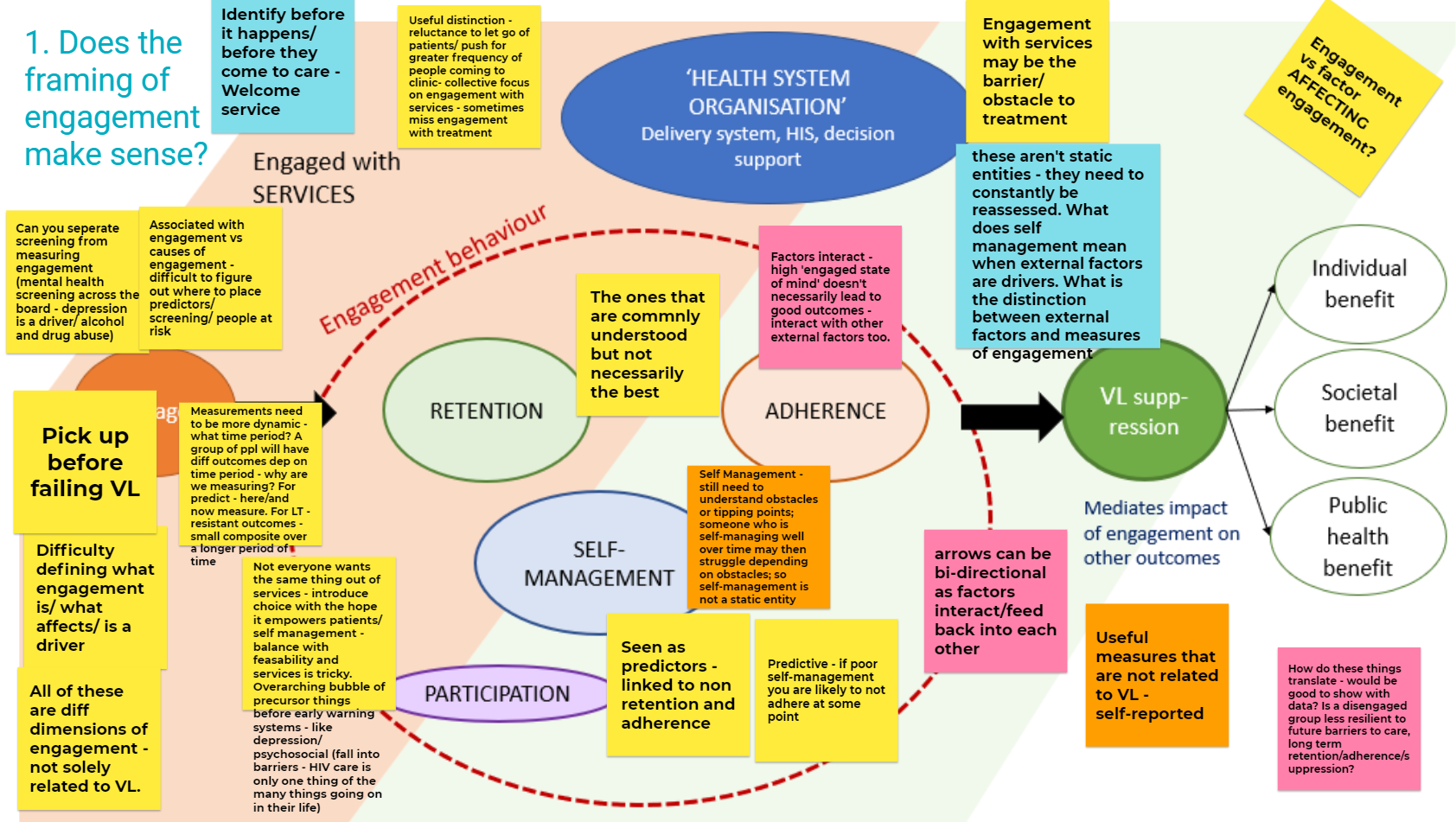


# References

1. Bright FAS, Kayes NM, Worrall L, McPherson KM. A conceptual review of engagement in healthcare and rehabilitation. *Disabil Rehabil*. 2015;37(8):643-654. doi:10.3109/09638288.2014.933899

2. Skovdal M, Wringe A, Seeley J, et al. Using theories of practice to understand HIV-positive persons varied engagement with HIV services: A qualitative study in six Sub-Saharan African countries. *Sex Transm Infect*. 2017;93(Supplement 3):52977. doi:http://dx.doi.org/10.1136/sextrans-2016-052977

3. Amico KR, Wallace M, Bekker L-G, et al. Experiences with HPTN 067/ADAPT Study-Provided Open-Label PrEP Among Women in Cape Town: Facilitators and Barriers Within a Mutuality Framework. *AIDS Behav*. 2017;21(5):1361-1375. doi:10.1007/s10461-016-1458-y

4. MacColl Center for Health Care Innovation. The Chronic Care Model. Improving Chronic Care.

5. Rivet Amico K. A situated-information motivation behavioral skills model of care initiation and maintenance (sIMB-CIM): An IMB model based approach to understanding and intervening in engagement in care for chronic medical conditions. *J Health Psychol*. 2011;16(7):1071-1081. doi:10.1177/1359105311398727

6. Swendeman D, Ingram BL, Rotheram-Borus MJ. Common elements in self-management of HIV and other chronic illnesses: an integrative framework. *AIDS Care*. 2009;21(10):1321-1334. doi:10.1080/09540120902803158.Common

7. World Health Organisation. *Innovative Care for Chronic Conditions: Building Blocks for Action*.; 2002.

8. Schulman-Green D, Jaser SS, Park C, Whittemore R. A Metasynthesis of Factors Affecting Self-Management of Chronic Illness. *J Adv Nurs (John Wiley Sons, Inc)*. 2016;72(7):1469-1489. doi:10.1111/jan.12902.A

9. Lorig KR, Holman HR, Med AB. Self-Management Education: History , Definition , Outcomes , and Mechanisms. *Ann Behav Med*. 2003;26(1):1-7.

10. Wagner EH. Organizing Care for Patients With Chronic Illness Revisited. *Milbank Q*. 2019;97(3):659-664. doi:10.1111/1468-0009.12416

11. Wagner EH, Austin BT, Korff M Von, Wagner EH, Austin BT. Organizing Care for Patients with Chronic Illness Published by : Wiley on behalf of Milbank Memorial Fund Stable URL : http://www.jstor.org/stable/3350391 Linked references are available on JSTOR for this article : 3 . Organizing Care for Patients with Ch. *Milbank Q*. 1996;74(4):511-544.

12. Blue S, Shove E, Carmona C, et al. Theories of practice and public health: understanding ( un ) healthy practices. *Crit Public Health*. 2016;26(1):36-50. doi:10.1080/09581596.2014.980396

13. McMillan SS, King M, Tully MP. How to use the nominal group and Delphi techniques. *Int J Clin Pharm*. 2016;38(3):655-662. doi:10.1007/s11096-016-0257-x

14. Arksey H, O’Malley L. Scoping studies: Towards a methodological framework. *Int J Soc Res Methodol Theory Pract*. 2005;8(1):19-32. doi:10.1080/1364557032000119616

15. Levac D, Colquhoun H, O’Brien KK. Scoping studies: Advancing the methodology. *Implement Sci*. 2010;5(1):1-9. doi:10.1186/1748-5908-5-69

16. Waggoner J, Carline JD, Durning SJ. Is there a consensus on consensus methodology? Descriptions and recommendations for future consensus research. *Acad Med*. 2016;91(5):663-668. doi:10.1097/ACM.0000000000001092

17. Keene CM, Ragunathan A, Euvrard J, et al. Measuring patient engagement with HIV care in sub-Saharan Africa: a scoping study. *JIAS*. 2022;25(10):e26025. doi.org/10.1002/jia2.26025
